# Long-term (10-year) monitoring of transposon-mediated transgenic cattle

**DOI:** 10.1007/s11248-024-00401-0

**Published:** 2024-08-28

**Authors:** Soo-Young Yum, Bae Young Choi, Gyeong-Min Gim, Kyeong-Hyeon Eom, Seong-Beom Lee, Daehyun Kim, Euntaek Lim, Do-Yoon Kim, Seong-Eun Heo, Donghwan Shim, Goo Jang

**Affiliations:** 1 LARTBio Inc., Gwangmyeong-si, Gyeonggi-do Republic of Korea; 2 Department of Biological Sciences, Chungnam National University, 99 Daehak-ro, Yuseong-gu, Daejeon, Republic of Korea; 3 Laboratory of Theriogenology and Biotechnology, Department of Veterinary Clinical Science, College of Veterinary Medicine and the Research Institute of Veterinary Science, Seoul National University, Gwanak-go, Gwanak-ru, Seoul, Republic of Korea; 4 Department of Animal Science, Chonnam National University, Gwangju, Republic of Korea; 5 Theragen Bio Co., Ltd., Seongnam, Gyeonggi-do Republic of Korea; 6 Gyeongsangbukdo Livestock Research Institute, Yeongju, Republic of Korea; 7 Center for Genome Engineering, Institute for Basic Science, Daejeon, Republic of Korea; 8 Comparative Medicine Disease Research Center, Seoul National University, Seoul, Republic of Korea

**Keywords:** Transposon, Germline transmission, Transgenic cattle, Long-term (ten-years), Genome stability, Somatic mutation

## Abstract

The production of transgenic animals using non-viral methods has raised questions regarding their long-term health and genomic stability. In this study, we evaluated these aspects in transgenic cattle over ten years, using transposon-mediated gene transfer. Our longitudinal analysis included a comprehensive health assessment and whole-genome DNA resequencing. We found no significant alterations in physiological parameters or health complications in transposon-mediated transgenic cattle that exceeded 10 years of age. Genomic analysis revealed that the rates of somatic mutations and copy number variations in transgenic cattle were comparable to those in non-transgenic cattle. Furthermore, structural variants were infrequent, suggesting that transposon-mediated gene insertion did not compromise genomic integrity. These findings highlight the viability of transposon systems for generating transgenic livestock, potentially expanding their applications in agriculture and biotechnology. This study contributes significantly to our understanding of the long-term implications of transgenesis in large animals and supports the safety and stability of this method.

**Supplementary Information:**

The online version contains supplementary material available at 10.1007/s11248-024-00401-0.

## Introduction

Transgenesis research in large animals has faced significant challenges since its inception in 1985 (Fischer and Schnieke 2023; Yang et al. 2021). Injecting plasmids into in vitro-fertilized oocytes and transferring embryos expressing them to produce the desired traits is thought to be useful for a variety of studies, including breed improvement, characterization of gene function, and disease resistance and related studies have been proven primarily in rodents. However, studies on large animals have not progressed as quickly as expected.

The advent of Dolly, a cloned lamb, resulted in a major shift in large animal studies using somatic cell nuclear transfer (Campbell et al. 1996). Gene engineering in pigs with relatively short gestational periods and large litter numbers has undergone notable advancements. Conversely, scientific progress in gene engineering of cattle has been slow because of the longer gestation period and production of single offspring (Yang et al. 2021). Moreover, owing to its high cost, there are difficulties in developing technologies to produce transgenic cattle. In contrast to pig research, the validity of long-term research findings, such as cattle gene modification and germline transmission in altered animals, is rather limited. Studies of germline transfer are scarce (Gim and Jang 2024; Gim et al. 2020; Reichenbach et al. 2010; Shakweer et al. 2023; Young et al. 2020; Yum et al. 2018).

Previously, we successfully produced cattle expressing green fluorescent protein (GFP) and yellow fluorescent protein (YFP) and conducted research on embryos up to the F2 generation. Subsequently, we proceeded to observe and assess the health and growth of the animals (Yum et al. 2018). Hence, in our investigation, the eldest animal exceeded 10 years of age and thrived in good health. Additionally, animals born thereafter are presently 9 and 7 years old, respectively. Consequently, we present health monitoring data and examine the genomic stability of these animals. Because genomic stability is an important aspect of regulatory review before genetically engineered animals can be approved, we report on the genetic status of these older animals.

## Materials and methods

### Animals

The genetically engineered animals analyzed in this report are those in Yum et al. 2016 (SNU-SB-1: 9 years and 8 months old, SNU-PB-1: 9 years and 1 month old, and SNU-F1-1: 7 years old).

### Blood analysis biosensor-based animal monitoring

To evaluate the general health status of the three transgenic cattle (SNU-SB-1: 9 years and 8 months old, SNU-PB-1: 9 years and 1 month old, and SNU-F1-1: 7 years old), 5 mL of whole blood was collected from the jugular vein for complete blood count analysis (Hemavet 950; Drew Scientific, USA) and serum chemistry (BS-400; Mindray, China) analyses. The animals were monitored using a biosensor (SmaXtec), and a veterinarian assessed their general health conditions.

### Library construction and sequencing

Genomic DNA was extracted from the blood of the cattle (SNU-SB-1: 10 years old, SNU-PB-1: 9 years and 5 months old and WT: 9 years and 9 months old). The quality and quantity of purified DNA were assessed using fluorometry (Qubit, Invitrogen) and gel electrophoresis. Briefly, 100 ng of genomic DNA from each sample was fragmented by acoustic shearing using a QSonica 800 R2 instrument. Fragments of 350 bp were ligated to the Illumina adapters and amplified. 500–600 bp is the appropriate size for the final library. Libraries were quantified using a TapeStation 4200 instrument (Agilent Technologies) and the KAPA Library Quantification Kit (KK4824, Kapa Biosystems). The resulting purified libraries were applied to an Illumina flow cell for cluster generation and sequenced using 150 bp paired-end reads on an Illumina NovaSeq 6000 (Illumina) sequencer according to the manufacturer’s protocols.

### Identification of somatic variants

Paired-end reads were cleaned using Trimommatic v 0.39 with the following parameters: LEADING 10, TRAILING 10, and MINLEN 50 (Bolger et al. 2014). Filtered reads were aligned to the reference *Bos taurus* genome (ARS UCD1.2, https://jul2023.archive.ensembl.org/Bos_taurus/Info/Index) using Burrows-Wheeler Aligner (BWA) v 0.7.17-r1188 (Li and Durbin 2010). The resulting SAM files were sorted using SAMtools v 1.19.2 (Danecek et al. 2021). Sample-level BAM files were subjected to variant calling using the GATK HaplotypeCaller pipeline. Variants from each sample were combined using the GATK CombineGVCFs pipeline, and genotypes were established using the GATK GenotypeGVCFs pipeline (O’Connor 2020). Variants located in the mitochondrial genome, X chromosome, and unanchored scaffolds were excluded from the analysis. Two types of somatic variants were assigned based on the following criteria: (type I) variants classified as a heterozygous genotype solely in 10-year-old transgenic cattle but classified as a reference homozygous genotype in 1-year-old transgenic cattle and wild-type (WT) controls and (type II) variants classified as a heterozygous genotype solely in 1-year-old transgenic cattle, but classified as a reference homozygous genotype in 10-year-old transgenic cattle and WT controls. To obtain high-confident variants, the somatic variants were filtered using BCFtools v 1.13 with the following parameters: depth >  = 10 and variant allele fraction >  = 0.35 (Danecek et al. 2021). The functional effects of the variants were predicted by applying SnpEff v 5.1d with ARS-UCD1.2 annotation (Cingolani et al. 2012).

### Analysis of somatic structural variants (SVs) and copy number alterations (SCNAs)

To identify SVs (deletions, duplications, inversions, and translocations) and SCNAs in transgenic cattle, we used Delly v 1.2.6 (Hubacek & Matouskova 1987). To obtain high-confidence SVs, the resulting SVs were filtered using BCFtools v 1.13, with the following parameters: PRECEISE = 1 and PE >  = 20 and MAPQ >  = 60 (Schurings 1987). Two types of somatic SVs were identified using the criteria described above. For SCNA analysis, we first detected SCNAs in 10-year-old transgenic cattle using 10 kbp mappable windows with coverage normalization. Subsequently, the resulting SCNAs detected in 10-year-old transgenic cattle were found in the 1-year-old transgenic cattle. The resulting SCNAs from both 1- and 10-year-old transgenic cattle were analyzed using BCFtools v 1.13 (Schurings 1987).

### Transgene insertion site detection using Oxford Nanopore long-read sequencing

High molecular weight DNA was isolated from the blood samples of 10-year-old transgenic cattle using the Wizard® HMW DNA Extraction Kit (A2920, Promega, USA) following the manufacturer’s instructions. Samples with a DNA integrity number > 9.0 were subjected to library preparation. Oxford Nanopore Technologies (ONT) libraries were constructed using a ligation sequencing kit (SQK-LSK110, ONT, UK) and subsequently sequenced on the ONT MinION platform according to the manufacturer’s instructions. Ratatosk v 0.7.6 was employed to correct errors in long reads using paired-end short reads (Holley et al. 2021). To identify the transgene insertion site within the chromosome of 10-year-old transgenic cattle, long reads were aligned to the transgene sequence using Minimap2 v 2.25 to isolate long reads containing the transgene sequence (Li 2018). The transgene insertion site was inferred by manual inspection using Integrative Genomics Viewer (Robinson et al. 2023) with SAM files, in which long reads containing the transgene sequence were aligned to the cow reference genome (ARS-UCD1.2) using Minimap2 v 2.25.

## Results

### Growth & health monitoring

**Fig. 1:**
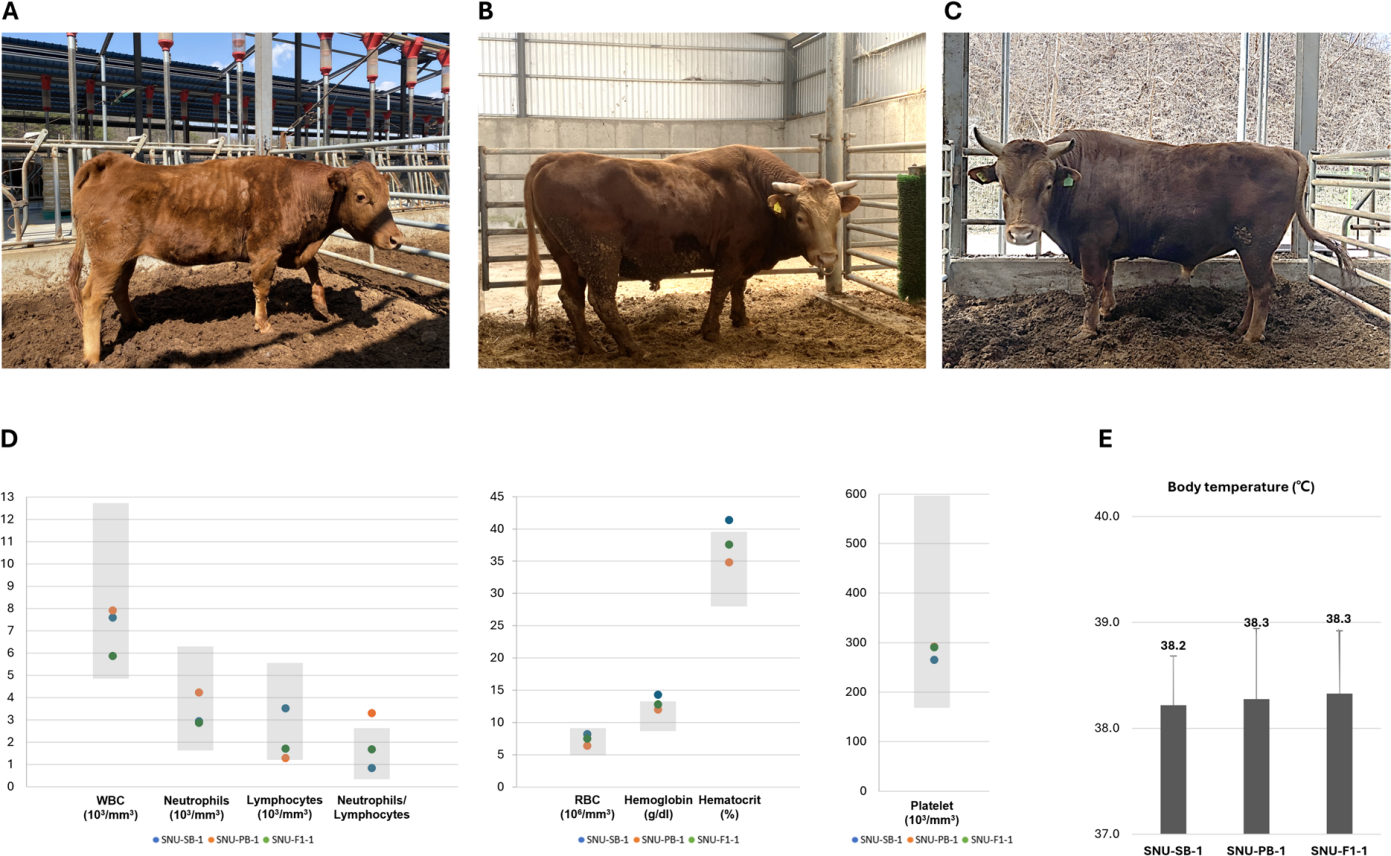
Monitoring health in transgenic cattle. Representative images of transgenic cattle **A** SNU-SB-1, **B** SNU-PB-1, and **C** SNU-F1-1. **D** Analysis of blood parameters from three transgenic cattle; WBC: White blood cells; RBC: Red blood cells; Gray box: reference range. **E** Average body temperature of individual transgenic cattle over 6 months

The F0 female transgenic cattle expressing YFP using Sleeping Beauty (SB) transposon system (SNU-SB-1) were born in December 2013 (Yum et al. 2016). She is now more than 10 years old (Fig. 1A) and has successfully delivered one calf naturally. Furthermore, her reproductive function was proven to be normal through a two-cycle ovum pick up, as assessed by ultrasound examination. Furthermore, she has been doing well without any notable health issues. The F0 male expressing GFP using *PiggyBac* (PB) transposon system (SNU-PB-1), as documented by Yum et al. in 2016, was born in August 2015 and is presently 9 years old (Fig. 1B). The individual exhibited typical ejaculation and successfully produced frozen semen. The offspring were conceived by in vitro fertilization (Gim et al. 2020) and is currently growing without further health complications. The male offspring expressing GFP (SNU-F1-1), resulting from the natural breeding of these two founders (SNU-SB-1 and SNU-PB-1) was produced to validate germline transmission and reproductive performance. This offspring was confirmed to have inherited only the PB-mediated GFP transgene from SNU-PB-1. SNU-F1-1 is currently seven years old and exhibits healthy growth, with no issues related to sperm collection or overall health (Fig. 1C) (Yum et al. 2018). The animal keeper assessed the essential characteristics of the animals, including their overall health, feed consumption, excretion, urine, and daily behavior. No significant changes were observed when compared with the other cattle.

**Fig. 2:**
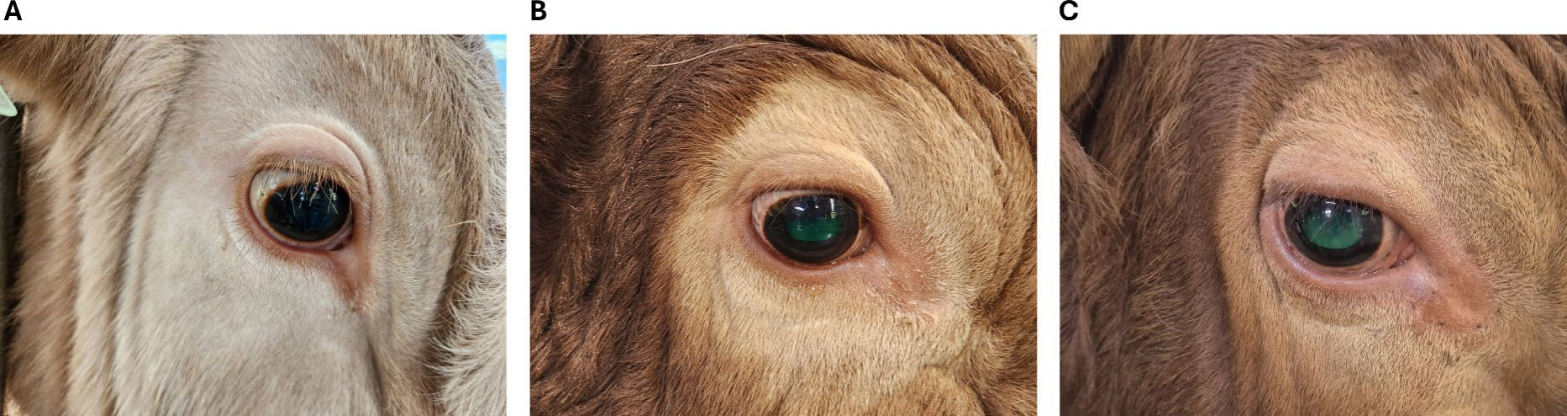
GFP expression in eye of transgenic cattle. Transgenic cattle showing a GFP expression in eye by PB transposon system. **A** WT, **B** SNU-PB-1, and **C** SNU-F1-1

In the irregular blood analysis, recent blood tests conducted in 2023 on all three cattle revealed no significant health issues and there were no differences compared to the control group (Fig. 1D). Additionally, by monitoring the body temperature of transgenic individuals over six months using biosensors, the collected data indicated that they maintained a normal body temperature (Fig. 1E). In the case of SNU-PB-1 and SNU-F1-1, stable GFP expression was observed in the eyes (Fig. 2).

### Genome stability for transposon-mediated transgenic cattle

**Fig. 3:**
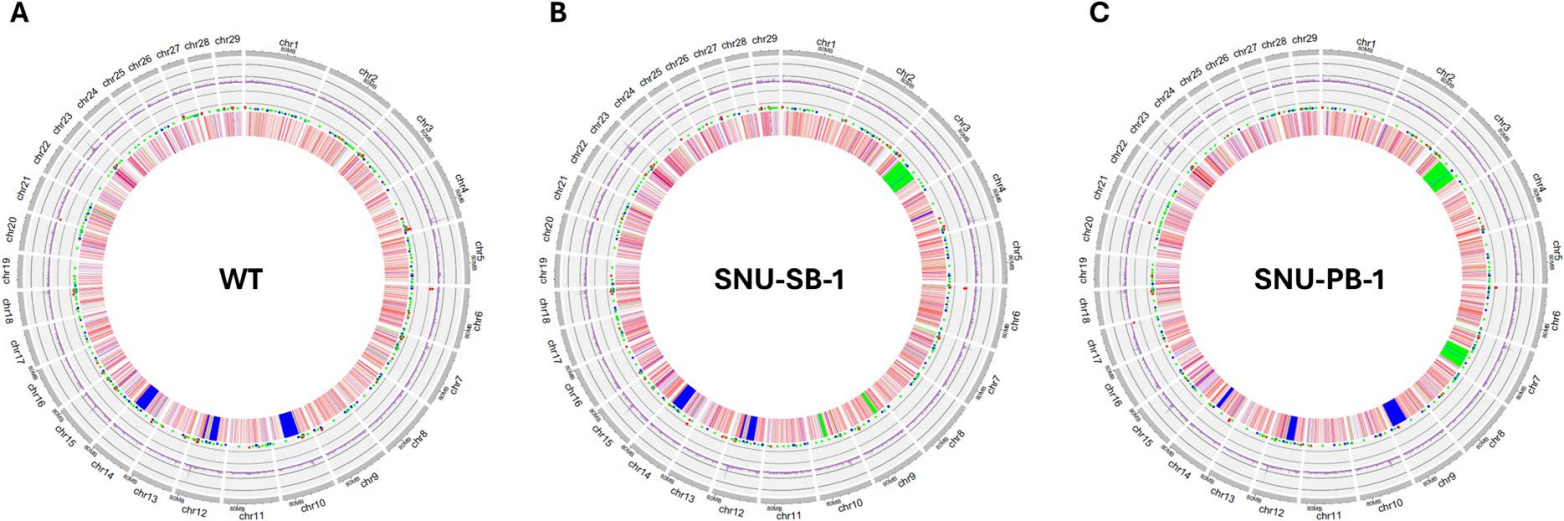
Overview of genomic variation in cattle. This figure presents an overview of genomic variation in cattle, with the following layers depicted from outer to inner: (1) chromosomes (chr) from chr1 to chr29 are depicted in peripheral grey boxes, based on the reference Bos Taurus genome (ARS UCD1.2), (2) the number of SNPs per 100-kb window is represented in a bar plot (purple), (3) CNVs in indicated positions are represented by dots, with colors indicating different CNV levels: CNV > 2 (red), CNV = 2 (green), and CNV < 2 (blue), (4) SVs are depicted, including deletion (red), inversion (blue), duplication (green), translocation (purple), and insertion (yellow). **A** 10-year-old WT, **B** 10-year-old SNU-SB-1, and **C** 9-year-old SNU-PB-1. (Color figure online)

To assess the effects of transposon-mediated transgene insertion on genome stability over a 10-year period (SNU-SB-1) and a 9-year period (SNU-PB-1), we performed whole-genome DNA resequencing using blood samples from transgenic cattle and an age-matched WT control. The average mapping rate of high-quality filtered reads to the cow reference genome (ARS-UCD1.2) exceeded 99.5%, with aligned paired-end reads covering a minimum sequencing depth of 11.9-fold across the entire genome for variant analysis (Table S1). In SNU-SB-1 and SNU-PB-1, the number of genomic variants detected was comparable to that in WT cattle (Fig. 3), suggesting that the presence of transgenes in transgenic cattle did not affect the overall genome structure over a 10-year period for SNU-SB-1 and a 9-year period for SNU-PB-1. We further integrated these data with our previously obtained results from 1-year-old transgenic cattle and an age-matched WT control to identify somatic variants over periods of 10 years (for SNU-SB-1) and 9 years (for SNU-PB-1). Using these data, we investigated somatic variants in 10-year-old and 9-year-old transgenic cattle relative to their 1-year-old transgenic counterparts and WT controls (Cagan et al. 2022).

We identified somatic single-nucleotide polymorphisms (SNPs) and insertions and deletions (Indels) in over 9–10 years for SNU-SB-1 and SNU-PB-1. SNU-SB-1 and SNU-PB-1 contained 6,155 and 7,990 somatic SNPs and 3,367 and 3,652 somatic Indels, respectively (Table 1). Among these variants, 17 and 9 non-reference homozygous (NonRefHom) SNPs were found in SNU-SB-1 and SNU-PB-1 cattle, respectively, along with 132 and 111 NonRefHom Indels. Notably, all somatic NonRefHom variants in transgenic cattle were located in non-coding regions, with their predicted functional impact categorized as “modifier” (Table S2-S5), indicating no discernible alteration in the function of protein-coding genes. Furthermore, the number of somatic variants detected in 10-year-old transgenic cattle was comparable to the previously reported non-transgenic somatic mutation rates in mammals (Cagan et al. 2022).

**Table 1:** Statistics of high-confidence somatic SNP and INDEL in transgenic cattle compared to WT. Somatic SNP and INDEL were detected using GATK v 4.3.0.0. with the ARS UCD1.2 cattle genome as the reference

ID	Type	Sample	Genotype	Number of SNPs	Number of INDELs
SNU-SB-1	Somatic variants type I	10 yr WT	Ref Hom	5305	2889
		10 yr transgenic cattle	Alt		
		1 yr WT	Ref Hom		
		1 yr transgenic cattle	Ref Hom		
	Somatic variants type II	10 yr WT	Ref Hom		478
		10 yr transgenic cattle	Ref Hom		
		1 yr WT	Ref Hom		
		1 yr transgenic cattle	Alt		
SNU-PB-1	Somatic variants type I	10 yr WT	Ref Hom	5771	2825
		10 yr transgenic cattle	Alt		
		1 yr WT	Ref Hom		
		1 yr transgenic cattle	Ref Hom		
	Somatic variants type II	10 yr WT	Ref Hom	2219	827
		10 yr transgenic cattle	Ref Hom		
		1 yr WT	Ref Hom		
		1 yr transgenic cattle	Alt		

Next, we identified SVs in 10-year-old transgenic cattle and compared them with WT controls. Six and two somatic SVs were detected in SNU-SB-1 and SNU-PB-1 cattle, respectively (Table 2), all of which were heterozygous. Prediction of the impact of SVs revealed transcriptional ablation of two genes in SNU-SB-1 cattle, ENSBTAG00000037925 and FNIP1 (Table S6), whereas no such effect was observed in SNU-PB-1 cattle (Table S7). The copy number variations (CNVs) in 10-year-old transgenic cattle mirrored those detected in 1-year-old transgenic cattle, indicating the absence of somatic copy number alterations over 10 years in transgenic cattle (Tables S8 and S9). Taken together, our whole-genome DNA resequencing data indicated that transposon-mediated transgene insertion in transgenic cattle did not perturb genome stability over the course of a decade.

**Table 2:** Statistics of high-confidence somatic Structure Variant (SV) in transgenic cattle compared to WT. Somatic SV were detected using Delly v1.2.6. with the ARS UCD1.2 cattle genome as the reference

ID	Type	Sample	Genotype	Number of SVs
SNU-SB-1	Somatic variants type I	10 yr WT	Ref Hom	6
		10 yr transgenic cattle	Alt	
		1 yr WT	Ref Hom	
		1 yr transgenic cattle	Ref Hom	
	Somatic variants type II	10 yr WT	Ref Hom	0
		10 yr transgenic cattle	Ref Hom	
		1 yr WT	Ref Hom	
		1 yr transgenic cattle	Alt	
SNU-PB-1	Somatic variants type I	10 yr WT	Ref Hom	2
		10 yr transgenic cattle	Alt	
		1 yr WT	Ref Hom	
		1 yr transgenic cattle	Ref Hom	
	Somatic variants type II	10 yr WT	Ref Hom	0
		10 yr transgenic cattle	Ref Hom	
		1 yr WT	Ref Hom	
		1 yr transgenic cattle	Alt	

### Identification of transgene insertion site using long-read sequencing and PCR analysis

To identify the transgene integration sites in SNU-SB-1 and SNU-PB-1, Oxford Nanopore long-read DNA sequencing was performed using the blood samples obtained from transgenic cattle. The average number of sequenced long reads exceeded 1,500,000, with an average N50 value of 39 kbp and a sequencing depth of approximately 11-fold (Table S10). Following the alignment of long-read sequences containing the transgene sequence (Fig. S1), we identified the integration sites of the transgene cassette within the genome of the transgenic cattle. In SNU-SB-1 cattle, the YFP gene cassette was integrated into chromosomes 4, 21, and 26. Two of these integrations were located within intron region and one was situated in an intergenic region (Table S11).

In SNU-PB-1 cattle, the GFP gene cassette was integrated into chromosomes 5 and 22 (Table 3). In comparison with our previous results (Yum et al. 2016), long-read sequencing analysis revealed that only 2 of the 15 GFP gene cassettes integrated into the genome of SNU-PB-1 were identified. The missing positional information for the other 13 GPF gene cassettes using long-read sequencing may be due to insufficient sequencing depth and the reduced copy number of GFP cassettes in the blood of SNU-PB-1 caused by somatic mosaicism. To verify the presence of undetected GFP gene cassettes in SNU-PB-1 cattle, we conducted PCR analysis to amplify the border sequence of the GFP gene cassettes using primer sets from our previous study (Yum et al. 2016). All 15 GFP gene cassettes integrated into the SNU-PB-1 genome were successfully amplified using PCR (Fig. S2), indicating the presence of 15 GFP gene cassettes in 9-year-old transgenic SNU-PB-1 cattle. Eight of the GFP cassettes were integrated in intergenic regions, seven were located within introns, and one was located downstream of *ENSBTAG00000039664* gene (Table S12). Except for the GFP cassette located downstream of *ENSBTAG00000039664* gene, the other GFP cassettes are unlikely to alter the expression of neighboring genes. Our combined long-read sequencing analysis and PCR validation demonstrated the robustness and stability of transposon-mediated gene delivery for over a decade.

**Table 3:** Integration sites validated by long-read sequencing data in transgenic cattle

	No	Chromosome	Insertion site	Annotation	Gene Name
SNU-SB-1	1	4	41,026,786–41,026,787	intron_variant	GNAI1
	2	21	27,884,710–27,884,711	intron_variant	ENSBTAG00000025764
	3	26	48,056,370–48,056,371	intergenic_region	ENSBTAG00000052677-ENSBTAG00000053738
SNU-PB-1	1	5	4,603,847–4,603,848	intergenic_region	ENSBTAG00000054123-CAPS2
	2	22	49,110,315–49,110,316	downstream_gene_variant	ENSBTAG00000039664

## Discussion

This study observed newborn transgenic cows for up to 10 years and evaluated their health and growth safety. To date, several transgenic cows, pigs, and sheep have been born, but most have reported the results of birth and germline transmission. The lifespan and health of transgenic animals remains a critical concern. Therefore, we raised cows born 10 and 9 years ago using the transposon system (Yum et al. 2016), and evaluated the presence or absence of abnormalities. Additionally, a study was conducted on the health and growth of animals born to them (Yum et al. 2018). In this study, two findings were obtained from the investigation of transgenic animals.

First, the YFP and GFP genes had no effect on the development or health of transgenic cattle born using a non-viral delivery method of a fluorescent gene. Furthermore, the transgene was not silenced, but was expressed continuously (Fig. 2). In other words, even if we use cells derived from transgenic animals, we can continuously track the cells for research purposes, and because there is no silencing, they can be used as an excellent research resource. As sperm are currently frozen, it is thought that they can be shared with people conducting related research.

Second, genome stability was analyzed. In economically valuable animals, such as cattle, it is difficult to study genomic mutations in older specimens. In this study, we conducted a cumulative analysis of genomic alterations in transgenic cattle, both one year and a decade later. From a safety perspective, the long-term health and normal physiological functions of transgenic animals produced using transposons underscore the lack of deleterious effects associated with transgene insertions. Indeed, our bioinformatic analyses using transgenic cattle demonstrated minimal perturbation in genome stability for over a decade (Fig. 3, Tables 1–3), suggesting that the introduction of transgenes via transposons rarely disrupts the overall genomic integrity of mammals during long-term development. These findings are consistent with the absence of harmful health effects in transgenic cattle (Fig. 1). The absence of health problems for over a decade is a robust indicator of the well-being of transgenic animals and the non-disruptive nature of transposon-mediated gene insertions. This prolonged period of health stability is critical for the application of such transgenic animals in the industry, where long-term productivity and sustainability are essential. In addition, when SNU-F1-2 was produced and slaughtered at the age of 38 months and nutritional analysis was conducted, no significant findings were observed in the nutritional analysis table (Table S13). In the future, we will analyze the health and genomic stability of SNU-F1-1 at 10 years of age.

Many previous studies have revealed that transgenic animals generated by pronuclear microinjection often exhibit mosaic patterns in their genomes (Burdon and Wall 1992; Chan et al. 1999; Kong et al. 2009). Genomic mosaicism in transgenic animals can lead to variegation in transgene copies across different cell types, resulting in diverse expression profiles of the transgenes. In our study, only 2 GFP transgene cassettes in SNU-PB-1 were detected by ONT sequencing (Table 3), but the remaining 13 were identified using PCR amplification (Fig. S2). The highly variable cell division rates in different cell types could facilitate the loss of transgenes (Kong et al. 2009), leading to somatic mosaicism of transgenes in transgenic animals. We speculated that, during the growth of SNU-PB-1, each transgene copy in hematopoietic stem cells might be gradually diluted due to the proliferation of non-transgenic cells. As a result, the GFP transgene cassettes in SNU-PB-1 were not easily detected by the less sensitive ONT sequencing, but most of them were identified using the more sensitive PCR methods.

In summary, our study provides evidence for the genomic stability and safety of transgenic animals produced using transposon systems. We monitored transposon-mediated transgenic cattle for more than 10 years and confirmed their normal health. Analysis of the reproduction of these animals was completed in our previous publication (Gim et al. 2020; Yum et al. 2016, 2018) and evidence has shown that even though the foreign genes (the fluorescent gene) has been expressed for 10 years, it has no particular effect on animal growth. Currently, we are producing genetically edited cattle and simultaneously conducting research on the health and safety of these animals (Gim et al. 2022, Gim et al. 2023). This study not only validates the use of transposon systems in large animal models but also opens the door for their broader application in agricultural biotechnology and genetic research.

## Supplementary Information

Below is the link to the electronic supplementary material.

Supplementary Figure S1. Integrative genomics viewer (IGV) visualization of long-read alignment in vector integration sites. (A) SNU-SB-1 and (B) SNU-PB-1. (PNG 491 KB)

Supplementary Figure S2. PCR analysis of exogenous gene insertion in transposon-mediated transgenic cattle. (A) SNU-SB-1 and (B) SNU-PB-1. (PNG 1219 KB)

Supplementary file3 (XLSX 203 KB)

## Data Availability

The datasets generated during the current study are available in the SRA database at the National Center for Biotechnological Information under the accession no. PRJNA1107024.

## Abbreviations

CNVs: Copy number variations
GFP: Green fluorescent protein
Indels: Insertions and deletions
ONT: Oxford nanopore technologies
PB: Piggybac
SB: Sleeping beauty
SCNAs: Copy number alterations
SNPs: Single-nucleotide polymorphisms
SV: Structure variant
SVs: Somatic structural variants
WT: Wild type
YFP: Yellow fluorescent protein
