# Supplementary figures and images for: Long-term (10-year) monitoring of transposon-mediated transgenic cattle
Source: Transgenic Res. 2024 Aug 28;33(5):503–12. doi: 10.1007/s11248-024-00401-0 (PMC11588892; doi:10.1007/s11248-024-00401-0)

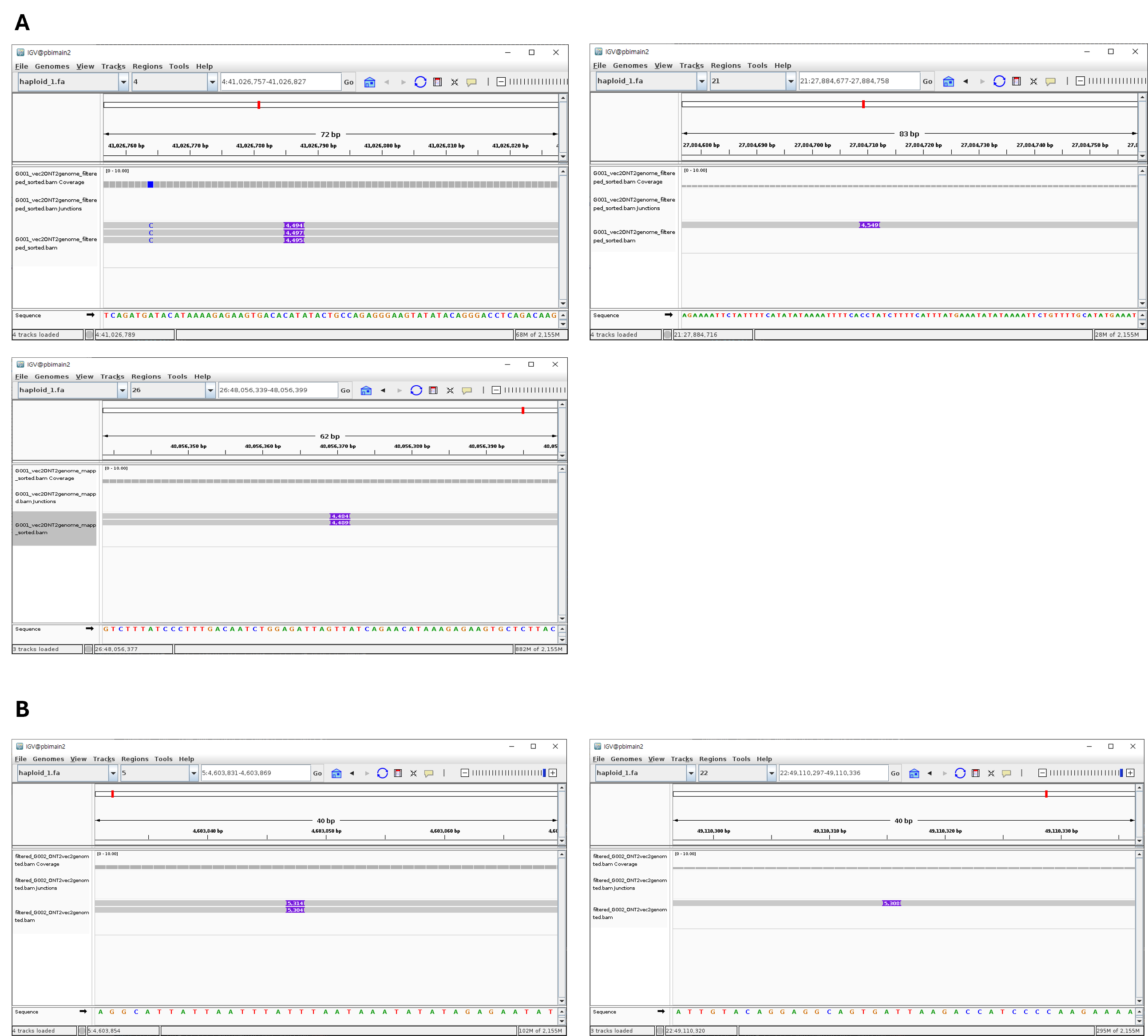

Supplement: Supplementary file 1 — Supplementary Figure S1. Integrative genomics viewer (IGV) visualization of long-read alignment in vector integration sites. (A) SNU-SB-1 and (B) SNU-PB-1. (PNG 491 KB) [file 11248_2024_401_MOESM1_ESM.png]

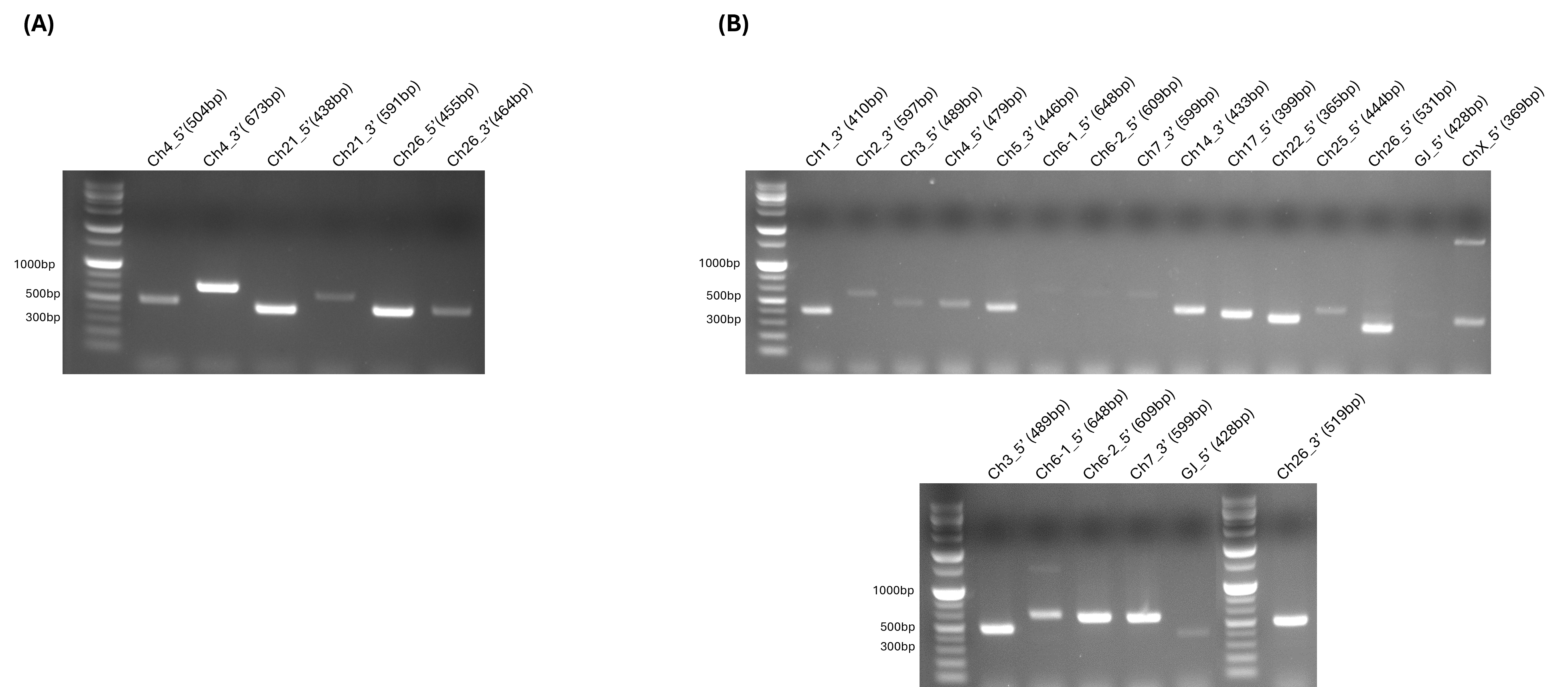

Supplement: Supplementary file 2 — Supplementary Figure S2. PCR analysis of exogenous gene insertion in transposon-mediated transgenic cattle. (A) SNU-SB-1 and (B) SNU-PB-1. (PNG 1219 KB) [file 11248_2024_401_MOESM2_ESM.png]
